# Diversity of RNA viruses in the cosmopolitan monoxenous trypanosomatid *Leptomonas pyrrhocoris*

**DOI:** 10.1186/s12915-023-01687-y

**Published:** 2023-09-12

**Authors:** Diego H. Macedo, Danyil Grybchuk, Jana Režnarová, Jan Votýpka, Donnamae Klocek, Tatiana Yurchenko, Jan Ševčík, Alice Magri, Michaela Urda Dolinská, Kristína Záhonová, Julius Lukeš, Elena Servienė, Alexandra Jászayová, Saulius Serva, Marina N. Malysheva, Alexander O. Frolov, Vyacheslav Yurchenko, Alexei Yu. Kostygov

**Affiliations:** 1 Faculty of Science, University of Ostrava, 710 00 Ostrava, Czech Republic; 2 University of Stockholm, Stockholm, Sweden; 3 Central European Institute of Technology, Masaryk University, 625 00 Brno, Czech Republic; 4 University Hospital in Ostrava, Ostrava, Czech Republic; 5 Institute of Parasitology, Biology Centre, Czech Academy of Sciences, 370 05 České Budějovice, Czech Republic; 6 Faculty of Science, Charles University, 128 44 Prague, Czech Republic; 7 Department of Veterinary Medical Sciences, Alma Mater Studiorum - University of Bologna, Ozzano Dell’Emilia, 40064 Bologna, Italy; 8 Department of Epizootiology, Parasitology and Protection of One Health, University of Veterinary Medicine and Pharmacy, 041 81 Košice, Slovakia; 9 Faculty of Science, Charles University, BIOCEV, 252 50 Vestec, Czech Republic; 10 Division of Infectious Diseases, Department of Medicine, University of Alberta, Edmonton, AB T6G 2R3 Canada; 11 Faculty of Sciences, University of South Bohemia, 370 05 České Budějovice, Czech Republic; 12 Laboratory of Genetics, Institute of Botany, Nature Research Centre, 08412 Vilnius, Lithuania; 13 Institute of Parasitology, Slovak Academy of Sciences, 040 01 Košice, Slovakia; 14 University of Veterinary Medicine and Pharmacy, 041 81 Košice, Slovakia; 15 Department of Biochemistry and Molecular Biology, Institute of Biosciences, Vilnius University, 10257 Vilnius, Lithuania; 16 Zoological Institute of Russian Academy of Sciences, 199034 St. Petersburg, Russia

**Keywords:** Tombus-like viruses, Ostravirus, *Leishbuviridae*, *Qinviridae*, *Pyrrhocoris apterus*

## Abstract

**Background:**

Trypanosomatids are parasitic flagellates well known because of some representatives infecting humans, domestic animals, and cultural plants. Many trypanosomatid species bear RNA viruses, which, in the case of human pathogens *Leishmania* spp., influence the course of the disease. One of the close relatives of leishmaniae, *Leptomonas pyrrhocoris*, has been previously shown to harbor viruses of the groups not documented in other trypanosomatids. At the same time, this species has a worldwide distribution and high prevalence in the natural populations of its cosmopolitan firebug host. It therefore represents an attractive model to study the diversity of RNA viruses.

**Results:**

We surveyed 106 axenic cultures of *L. pyrrhocoris* and found that 64 (60%) of these displayed 2–12 double-stranded RNA fragments. The analysis of next-generation sequencing data revealed four viral groups with seven species, of which up to five were simultaneously detected in a single trypanosomatid isolate. Only two of these species, a tombus-like virus and an Ostravirus, were earlier documented in *L. pyrrhocoris*. In addition, there were four new species of *Leishbuviridae*, the family encompassing trypanosomatid-specific viruses, and a new species of *Qinviridae*, the family previously known only from metatranscriptomes of invertebrates. Currently, this is the only qinvirus with an unambiguously determined host. Our phylogenetic inferences suggest reassortment in the tombus-like virus owing to the interaction of different trypanosomatid strains. Two of the new *Leishbuviridae* members branch early on the phylogenetic tree of this family and display intermediate stages of genomic segment reduction between insect *Phenuiviridae* and crown *Leishbuviridae*.

**Conclusions:**

The unprecedented wide range of viruses in one protist species and the simultaneous presence of up to five viral species in a single *Leptomonas pyrrhocoris* isolate indicate the uniqueness of this flagellate. This is likely determined by the peculiarity of its firebug host, a highly abundant cosmopolitan species with several habits ensuring wide distribution and profuseness of *L. pyrrhocoris*, as well as its exposure to a wider spectrum of viruses compared to other trypanosomatids combined with a limited ability to transmit these viruses to its relatives. Thus, *L. pyrrhocoris* represents a suitable model to study the adoption of new viruses and their relationships with a protist host.

**Supplementary Information:**

The online version contains supplementary material available at 10.1186/s12915-023-01687-y.

## Background

Parasitic flagellates of the family Trypanosomatidae are a diverse group of protists, some of which are well-known pathogens of humans, domestic animals, and cultural plants [1]. According to their life cycle, they are subdivided into monoxenous (developing in one host) and dixenous (switching between two different hosts) species. Only a few genera, namely phytoparasitic *Phytomonas*, as well as vertebrate-infecting *Trypanosoma* and *Leishmania sensu lato* (i.e., including *Porcisia* and *Endotrypanum*) are dixenous, whereas the vast majority of known trypanosomatid lineages are monoxenous parasites of insects [2]. Arguably, the most diverse group within the family is the species-rich subfamily Leishmaniinae, which unites dixenous *Leishmania s*. *l*. and their closest monoxenous relatives of the genera *Borovskyia*, *Crithidia*, *Leptomonas*, *Lotmaria*, *Novymonas*, and *Zelonia* [3]. *Leptomonas pyrrhocoris* is one of the model species of this subfamily. It is an easily cultivable monoxenous parasite of the cosmopolitan and abundant firebug *Pyrrhocoris apterus* [4], with a genomic sequence assembled to a near-chromosome level [5].

Several species of *Leishmania* harbor double-stranded *Leishmania RNA viruses 1* and *2* (LRV1/2, genus *Leishmaniavirus*) of the family Totiviridae [6–8]. The distribution of these viruses into the LRV1 and LRV2 clades mirrors the separation of *Leishmania* into the New and Old World lineages, respectively, indicating a deep co-evolutionary history of leishmaniaviruses and their hosts [9, 10]. The LRV1 in *L*. *guyanensis* is responsible for more severe symptoms of mucocutaneous leishmaniasis caused by the virus interference with anti-*Leishmania* immune response, thereby promoting the survival of the infected macrophages [11–13].

The findings described above have inspired a broad survey of trypanosomatid viromes resulting in the discovery of several new viral groups [14]. These included viruses related to *Narnaviridae* and the new family *Leishbuviridae* (LBVs) broadly infecting trypanosomatids, as well as Leptomonas pyrrhocoris tombus-like virus (LeppyrTLV1) and Leptomonas pyrrhocoris Ostravirus (LeppyrOV1) restricted to *L. pyrrhocoris*. In addition, an endogenous viral element, homologous to the large segment of the LeppyrTLV1, was detected in the genome of *L*. *pyrrhocoris* suggesting a long-lasting interaction between the virus and the flagellate [14]. A representative of the trypanosomatid-specific family *Leishbuviridae* was also documented in a peculiar species of *Leishmania—L. martiniquensis* (subgenus *Mundinia*), in which it can modulate macrophage infection [15]. Interestingly, the LRV-related viruses were also found in *Blechomonas* spp., a divergent clade of monoxenous flea-infecting trypanosomatids, which apparently acquired the virus from *Leishmania* spp. [16].

Out of all trypanosomatid species studied to date, *L. pyrrhocoris* represents the most attractive model for studies of the diversity of RNA viruses because of its worldwide distribution, high prevalence in the natural firebug populations, and harboring of unique viruses. In this study, we broadened the scope of known viruses in *L. pyrrhocoris* by systematically sampling them across Europe. In addition to previously known Ostravirus and tombus-like viruses, we documented new divergent LBVs and a Qin-like virus, the latter being the first detection of this rare viral group in a trypanosomatid host.

## Results and discussion

### Infection prevalence and detection of double-stranded RNA

Out of 508 dissected firebugs, 374 (74%) were positive for trypanosomatids with prevalence varying between 27 and 100% for different localities. From this material, 106 axenic cultures of *L. pyrrhocoris* were established, of which 64 (60%) showed the presence of 2–12 double-stranded RNA (dsRNA) bands ranging from 1.3 to 6.2 kb in length (Table 1).

**Table 1:** Studied *Leptomonas pyrrhocoris* isolates: geographic origin and detection of viruses

**Country**	**City**	**Trypanosomatid prevalence**	**Positive for dsRNA/tested**	**Isolate**	**Fragments on the gel [kb]**	**Viral identity**	**NGS**
Belarus	Vitebsk	8/8 (100%)	1/3	BY-Vi257			
				BY-Vi260	3.5, 2.2	TLV	Yes
				BY-Vi262			
Czechia	Benešov	4/7 (57%)	0/2	CZ-Be02			
				CZ-Be04			
	Brno	7/10 (70%)	2/3	CZ-Br01	3.5, 2.2	TLV	
				CZ-Br02	3.5, 2.2	TLV, OV^a^, LBV1/2/4^a^	Yes
				CZ-Br07			
	České Budějovice	16/25 (64%)	0/4	CZ-CB02			
				CZ-CB03			
				CZ-CB13			
				CZ-CB16			
	Hradec nad Moravicí	12/16 (75%)	9/9	CZ-HM01	3.5, 2.2	TLV, LBV3^a^	Yes
				CZ-HM02	3.5, 2.2	TLV	Yes
				CZ-HM03	3.5, 2.2	TLV	
				CZ-HM04	3.5, 2.2	TLV	Yes
				CZ-HM05	3.5, 2.2	TLV	
				CZ-HM06	3.5, 2.2	TLV	Yes
				CZ-HM07	3.5, 2.3	TLV	
				CZ-HM08	3.5, 2.2	TLV	Yes
				CZ-HM09	3.5, 2.2	TLV	Yes
	Ostrava	24/30 (80%)	11/13	CZ-Os00	3.5, 2.2	TLV	
				CZ-Os01	3.5, 2.2	TLV	
				CZ-Os02	3.5, 2.2	TLV	
				CZ-Os03	3.5, 2.2	TLV	Yes
				CZ-Os05	3.5, 2.2	TLV	
				CZ-Os07	3.5, 2.2	TLV	
				CZ-Os08	3.5, 2.2	TLV	
				CZ-Os10	3.5, 2.2, 5, 4.2, 4, 3, 2.4, 1.6, 1.3	TLV, OV	
				CZ-Os11	3.5, 2.2	TLV	Yes
				CZ-Os12	3.5, 2.2	TLV	
				CZ-Os13			
				CZ-Os14			
				CZ-Os15	3.5, 2.2, 5.0, 4.2, 4.0, 3.0, 2.4, 1.6, 1.3	TLV, OV	
	Prague	12/21 (57%)	1/3	CZ-Pr02			
				CZ-Pr14	3.5, 2.2, 5.0, 4.2, 4.0, 3.0, 2.4, 1.6, 1.3, 6.2, 1.9, 1.5	TLV, OV, LBV3	Yes
				Cz-Pr24			
Germany	Jena	3/4 (75%)	1/1	DE-Je02	3.5, 2.2	TLV	Yes
Hungary	Varbó	10/16 (62%)	2/3	HU-Va04	3.5, 2.2	TLV	Yes
				HU-Va05	6.2, 1.9, 1.5	LBV3	Yes
				HU-Va09			
Italy	Grosseto	11/28 (39%)	2/3	IT-Gr06	3.5, 2.2	TLV	
				IT-Gr07	3.5, 2.2	TLV	
				IT-Gr10			
	Rome	8/30 (27%)	6/8	IT-Ro01	3.5, 2.2	TLV	Yes
				IT-Ro02	3.5, 2.2	TLV	
				IT-Ro03			
				IT-Ro04	3.5, 2.2	TLV	
				IT-Ro05			
				IT-Ro06	3.5, 2.2	TLV	Yes
				IT-Ro07	3.5, 2.2	TLV	
				IT-Ro08	3.5, 2.2	TLV	
Lithuania	Vilnius	8/8 (100%)	3/3	LT-Vi06	3.5, 2.2	TLV	Yes
				LT-Vi08	3.5, 2.2	TLV	Yes
				LT-Vi09	3.5, 2.2, 6.2, 1.9, 1.5	TLV, LBV3	Yes
Poland	Rzeszów	18/20 (90%)	3/6	PL-Rz05			
				PL-Rz06	3.5, 2.2, 5.0, 4.2, 4.0, 3.0, 2.4, 1.6, 1.3, 6.2, 1.9, 1.5	TLV, OV, LBV3	Yes
				PL-Rz11			
				PL-Rz12			
				PL-Rz13	3.5, 2.2	TLV	Yes
				PL-Rz18	6.2, 1.9, 1.5	LBV3	Yes
Portugal	Lisbon	6/13 (46%)	4/5	PT-Li02	3.5, 2.2	TLV	
				PT-Li03	3.5, 2.2	TLV	Yes
				PT-Li04	3.5, 2.2	TLV	
				PT-Li05			
				PT-Li06	3.5, 2.2, 6.2, 3.2, 1.8	TLV, LBV4	Yes
Romania	Luncavița	2/6 (33%)	1/1	RO-Lu01	3.5, 2.2	TLV	
Russia	Borisovka	8/8 (100%)	0/1	RU-Bo01			
	Krasnodar	11/11 (100%)	1/2	RU-Kr01	3.5, 2.2, 6.2, 1.9, 1.5	TLV, LBV3, OV^a^	Yes
				RU-Kr02			
	Moscow	14/17 (82%)	4/4	RU-Mo01	3.5, 2.2	TLV	Yes
				RU-Mo02	3.5, 2.2	TLV	Yes
				RU-Mo202	3.5, 2.2	TLV	
				RU-Mo203	3.5, 2.2	TLV	Yes
	Pskov	92/92 (100%)	0/3	RU-Ps01			
				RU-Ps02			
				RU-Ps03			
	Suyda	10/11 (91%)	1/1	RU-Su01	3.5, 2.2	TLV	Yes
Serbia	Dimitrovgrad	12/15 (80%)	2/4	SE-Dm02			
				SE-Dm03			
				SE-Dm04	3.5, 2.2	TLV	Yes
				SE-Dm07	3.5, 2.2, 6.2	TLV, LBV^b,c^	
	Subotica	10/13 (77%)	3/7	SE-Sb01			
				SE-Sb02	3.5, 2.2, 6.2, 1.9, 1.5	TLV, LBV2/3/4	Yes
				SE-Sb03			
				SE-Sb04			
				SE-Sb06	3.5, 2.2	TLV	
				SE-Sb07	3.5, 2.2, 6.2, 1.9, 1.5	TLV, QIN^a^, LBV2/3/4	Yes
				SE-Sb09			
Slovakia	Bratislava	25/30 (83%)	4/5	SK-Br02	3.5, 2.2, 6.2	TLV, LBV2^b^	Yes
				SK-Br05	3.5, 2.2	TLV	Yes
				SK-Br18	3.5, 2.2	TLV	
				SK-Br20	3.5, 2.2	TLV	
				SK-Br24			
	Košice	9/20 (45%)	0/1	SKK6			
	Liptovský Hrádok	7/10 (70%)	0/3	SK-LH01			
				SK-LH04			
				SK-LH06			
	Ľubochňa	18/25 (72%)	0/4	SK-Lu01			
				SK-Lu10			
				SK-Lu15			
				SK-Lu16			
Ukraine	Zaporizhzhia	9/14 (64%)	3/4	UA-Zp01	3.5, 2.2, 5.0, 4.2, 4.0, 3.0, 2.4, 1.6, 1.3	TLV, OV	Yes
				UA-Zp02	3.5, 2.2	TLV, OV^a^, QIN^a^	Yes
				UA-Zp03	3.5, 2.2	TLV	Yes
				UA-Zp04			
**Total**		**374/508 (74%)**	**64/106 (60%)**			**61 TLV, 13 LBV, 8 OV, 2 QIN**	**37**

^a^No fragments detected on the gel

^b^Only the large segment detected on the gel

^c^The viral species could not be identified in the absence of sequence data

### Next-generation sequencing and viral identification

Out of 64 dsRNA-containing isolates, 37 were selected for next-generation sequencing (NGS), with a focus on those displaying complex dsRNA patterns. These included samples from all countries except for Romania. The analysis of the sequence data allowed the identification of 58 viral genomes (Table 1), of which 32 belonged to the previously described LeppyrTLV1, 18 to four new species of the family *Leishbuviridae*, 6 to LeppyrOV1, and 2 to a new Qin-like virus. Each sequencing library contained 3.5–20 million reads resulting in the coverage ranging from 5 to 104 reads per kilobase per million (RPKM) for an individual viral genomic segment. The vast majority of viral sequences were complete with a few exceptions due to low coverage (the latter sequences were not deposited to GenBank). Complete genomes were assembled for all viruses, except for the clades 1 and 2 of leishbuviruses, in which the middle segments were not found. These glycoprotein-coding segments usually have an order of magnitude lower coverage compared to the large and small segments and highly divergent amino acid sequences, making the search extremely difficult. Fifteen positive isolates contained two to five viruses at once, suggesting coinfections.

### Tombus-like virus and Ostravirus

Out of 106 *L. pyrrhocoris* isolates tested, 8 and 61 displayed dsRNA bands consistent with the presence of LeppyrOV1 and LeppyrTLV1, respectively (Table 1). This was confirmed by the obtained genomic sequences for 6 LeppyrOV1 and 32 LeppyrTLV1 viruses. Sequence similarity to the previously described prototypical viruses [14] was quite high: the minimal nucleotide identity was 93% for tombus-like virus and 95% for Ostravirus. Open reading frames (ORFs) of LeppyrTLV1 and LeppyrOV1 contained 7.6–16.0% and 5.9–11.6% of variable sites at the nucleotide level or 3.5–6.8% and 1.4–12.8% of those at the amino acid level, respectively (Additional file 2: Table S2).

**Fig. 1:**
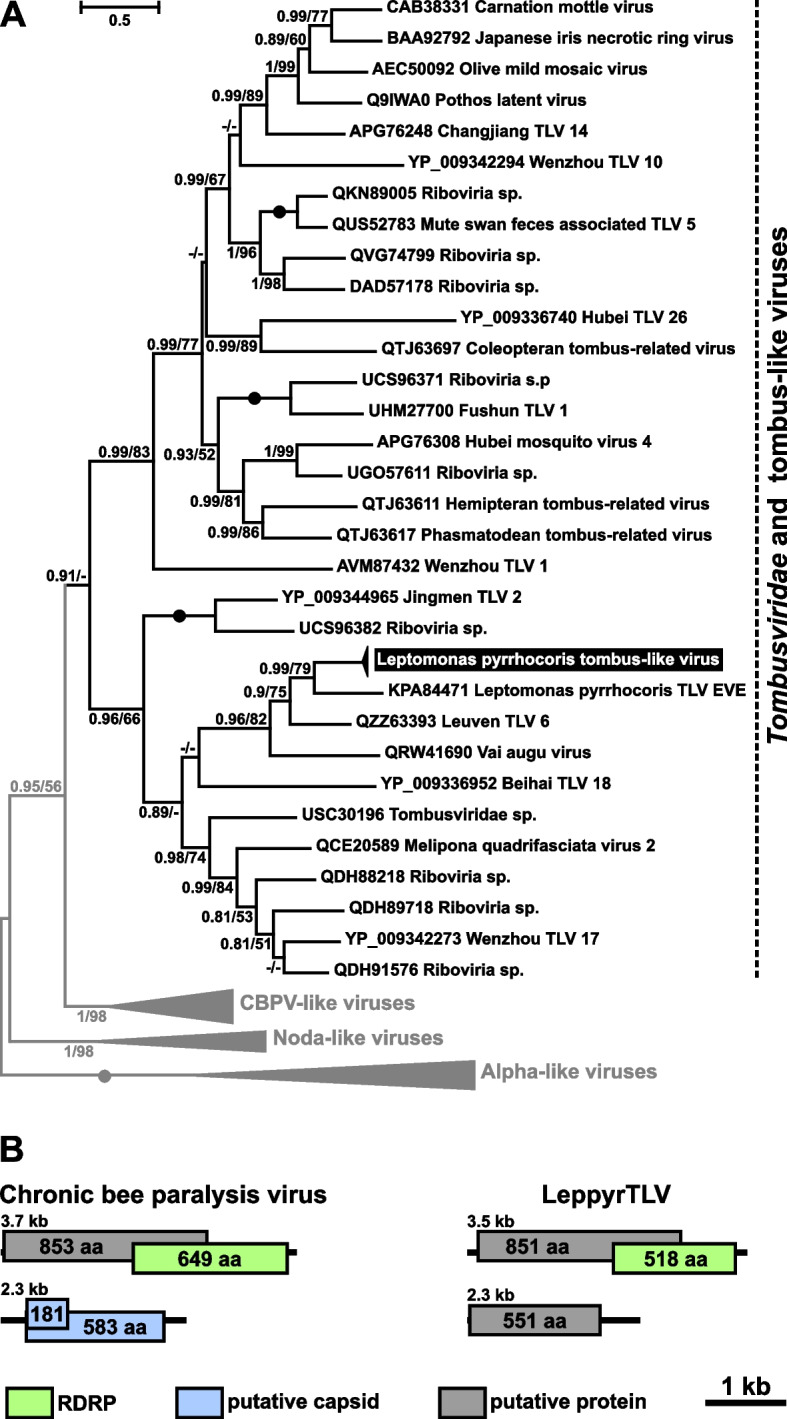
Tombus-like virus of *Leptomonas pyrrhocoris*. **A** ML phylogenetic tree based on RDRP amino acid sequences. Numbers at the branches indicate Bayesian posterior probability (PP) and ML bootstrap supports (BS). Only supports PP ≥ 0.8 and BS ≥ 50 are shown, lower values replaced with dashes (-). Circles correspond to maximal statistical support by both methods. The scale bar indicates the number of substitutions per site. Outgroups are shown in gray. Inversed font and background colors indicate the strains studied in this work. **B** Schemes of the genomic organization of LeppyrTLV1 and chronic bee paralysis virus, the closest available reference species

Phylogenetic analysis revealed that LeppyrTLV1 is related to two viruses recently detected in insects: Leuven tombus-like virus 6 from the common wasp *Vespula vulgaris* [17] and Vai augu virus from the tule mosquito *Culex erythrothorax* [18] (Fig. 1A). Previously, we proposed that LeppyrTLV1 could have originated from one of the viruses of non-insect invertebrates occasionally serving as food of firebugs [14]. The new data suggest that the ancestral virus could be of insect origin. Of note, it is not always clear, whether the viruses found in metatranscriptomes of insects actually belong to the latter and not to their microbiota, such as trypanosomatids. Indeed, wasps have been recorded as trypanosomatid hosts [19]. Moreover, as predators, they can temporarily contain non-specific parasites acquired from their insect prey. As for the mosquito virus, no trypanosomatids were detected in the Vai augu virus-containing samples [18].

The genome of LeppyrTLV1 contains two segments (Fig. 1B). The larger one (3.5 kb) carries two overlapping ORFs coding for an unidentified protein and for RNA-dependent RNA polymerase (RDRP). The smaller fragment (2.2 kb) comprises a single ORF for a putative protein. This organization is similar to that of the chronic bee paralysis virus (CBPV), which is the closest relative of LeppyrTLV1 with a known genomic structure. The length of the segments and ORFs are similar in both viruses, but the small segment of CBPV contains an additional small ORF overlapping with the main one and both are predicted to code for capsid proteins [20]. This suggests that the small segment of LeppyrTLV1 can also code for a capsid, although all our attempts to find homology between the proteins of these two viruses failed.

**Fig. 2:**
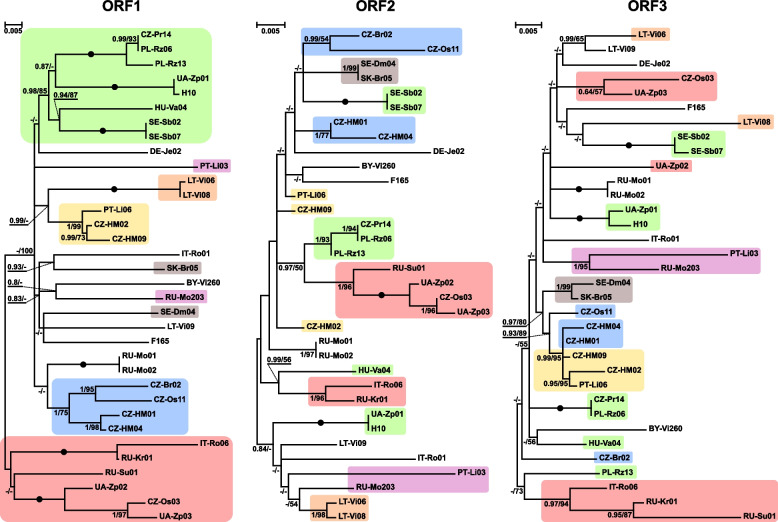
ML phylogenetic trees of the three ORFs of LeppyrTLV1 strains based on nucleotide sequences. Numbers at the branches indicate Bayesian posterior probability (PP) and ML bootstrap supports (BS). Only supports PP ≥ 0.8 and BS ≥ 50 are shown, lower values are replaced with dashes. Circles correspond to maximal statistical support by both methods. The scale bar indicates the number of substitutions per site. Some groups are highlighted for easier comparison of topologies

Phylogenetic relationships among individual LeppyrTLV1 viruses were rather poorly resolved, although some clades could be identified (Fig. 2). Importantly, the inferences using different ORFs produced conflicting topologies, and according to likelihood ratio test and Bayes factors analysis, their incompatibility was statistically highly significant (Additional file 3: Table S3). Such topological discordance suggests a reassortment of LeppyrTLV1 genomes, which may occasionally occur during mixed infections by different viral strains and is facilitated by their multi-segment organization. Considering the observed high prevalence of this virus in *L. pyrrhocoris* (57.5%), such mixed infections are very likely. The lack of strict phylogeographic structure in the inferred trees (i.e., viruses from a single location are not always most closely related to each other) (Fig. 2) suggests some intermixture of viral strains between geographic areas. Since neither LeppyrTLV1 nor its trypanosomatid host *L. pyrrhocoris* possesses long-lived stages able to travel over long distances themselves, the intermixture must be due to firebugs’ dispersal.

**Fig. 3:**
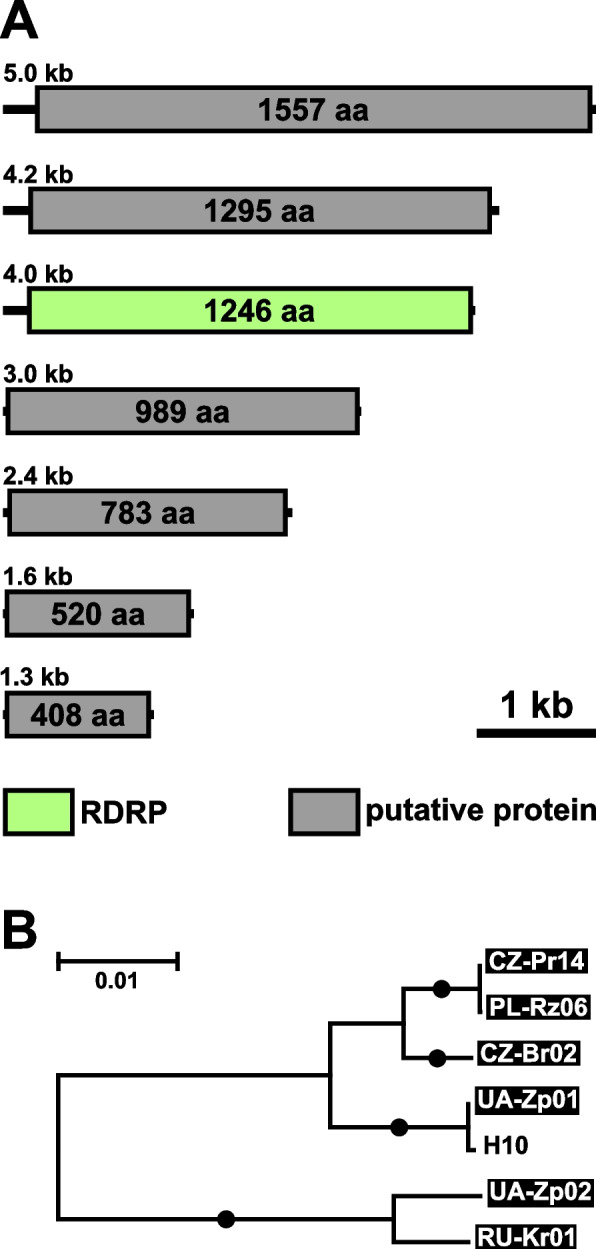
Ostravirus of *Leptomonas pyrrhocoris*. **A** Genomic organization of LeppyrOV1. **B** ML phylogenetic tree of LeppyrOV1 based on the concatenated nucleotide sequences of the seven ORFs. Circles correspond to maximal Bayesian posterior probability and ML bootstrap supports. The scale bar indicates the number of substitutions per site. Inversed font and background colors indicate the strains studied in this work

The NGS approach used here allowed identifying a previously overlooked 1.27-kb-long 7th segment of Ostravirus based on the specific 5′-terminal AAAGAAAAAAC sequence (Fig. 3A). Thus, the total length of this viral genome is 21.5 kb, a rather large size for an RNA virus. Confirming our previous assumption on the satellite nature of Ostravirus, it was invariably detected with LeppyrTLV1 [14]. It is surprising that a virus with such a large and complex genome cannot be self-sufficient. Yet, there are similar examples among DNA viruses: the members of *Lavidaviridae* (also known as virophages) have genome sizes comparable to that of Ostravirus (17–30 kb, 20 predicted ORFs) and can develop in their protist hosts only in the presence of giant viruses of the family *Mimiviridae* [21]. The relationships between LeppyrTLV1 and LeppyrOV1 appear unbalanced as judged by the ratios of their RPKM values in different samples ranging from nearly equal to over two orders of magnitude preponderance for the former (Additional file 1: Table S1), leading to failure of detection of the latter in the gel (Table 1). It appears plausible that only a minority of cells are infected in the isolates with a very low content of Ostravirus.

The phylogenetic tree reconstructed from the concatenated nucleotide alignment of all seven ostraviral ORFs was fully resolved into two major clades containing CZP02 and RUK02 (Czechia and Russia) and CZP01, CZBR02, CZP14, and PLE06 (Czechia, Ukraine, and Russia) with no apparent phylogeographic structure (Fig. 3B).

### Leishbuviridae

**Fig. 4:**
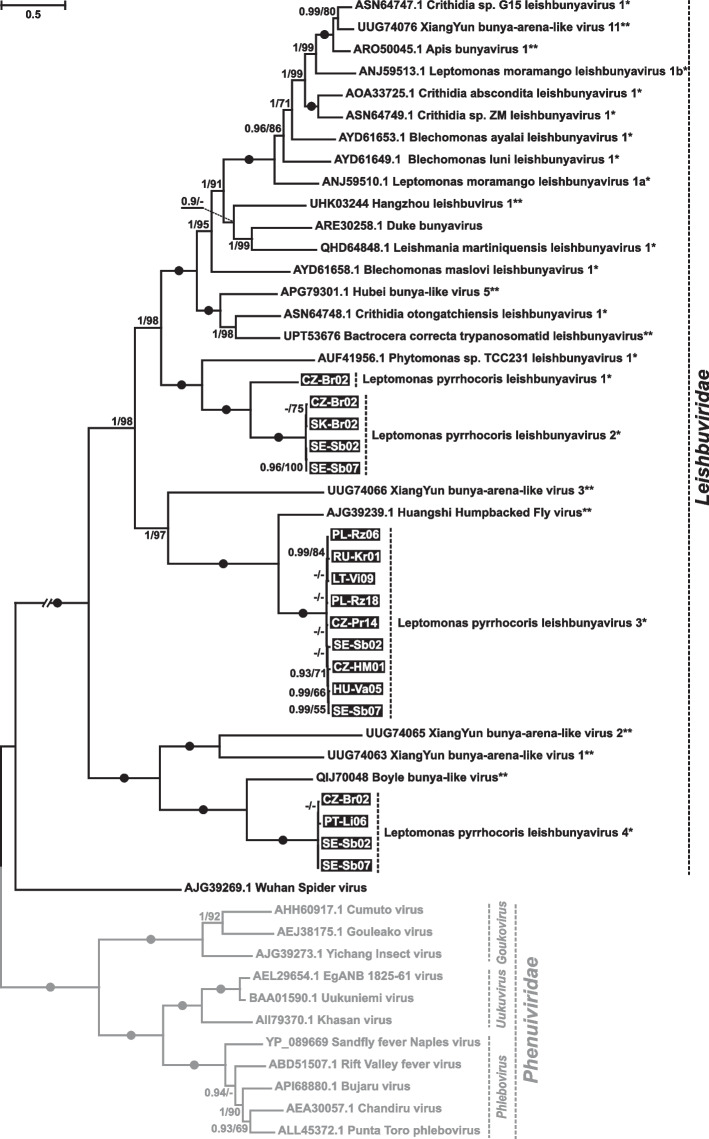
ML phylogenetic tree of Leishbuviridae based on RDRP amino acid sequences. Numbers at the branches indicate Bayesian posterior probability (PP) and ML bootstrap supports (BS). Only supports PP ≥ 0.8 and BS ≥ 50 are shown, lower values are replaced with dashes. Circles correspond to maximal statistical support by both methods. The scale bar indicates the number of substitutions per site. The double-crossed branch is at 50% of the original length. Outgroup is shown in gray. Inversed font and background colors indicate the strains studied in this work. Single asterisk marks viruses detected in trypanosomatid cultures, and two asterisks indicate viruses found in metatranscriptomes along with trypanosomatids (detected previously in [14] or reported in a corresponding NCBI Sequence Read Archive record in the “Taxonomy Analysis” section)

The family *Leishbuviridae* (previously, *Leishbunyaviridae*) is a speciose group of negative-sense single-stranded RNA (-ssRNA) viruses commonly infecting various lineages of trypanosomatids [14–16]. Here, we identified four novel representatives of this family in *L*. *pyrrhocoris*, which has not been previously recorded as a host of viruses from this group [14]. Interestingly, these viruses, named LeppyrLBV1–LeppyrLBV4 (for Leptomonas pyrrhocoris leishbuvirus 1 to 4), are not closely related to LBVs from other members of the subfamily Leishmaniinae (Fig. 4). Two new members of LBVs, namely LeppyrLBV1 and LeppyrLBV2, represent sister taxa with their closest relative being the virus from a plant-infecting trypanosomatid *Phytomonas* sp. TCC231. Some viral sequences previously reported from insect metatranscriptomes [22] proved to be nested within trypanosomatid LBVs (Fig. 4) suggesting that their hosts are also these flagellates. Indeed, one of these is a Huangshi Humpbacked Fly virus, identified in a fly, which, as we previously revealed, harbored trypanosomatids [14].

**Fig. 5:**
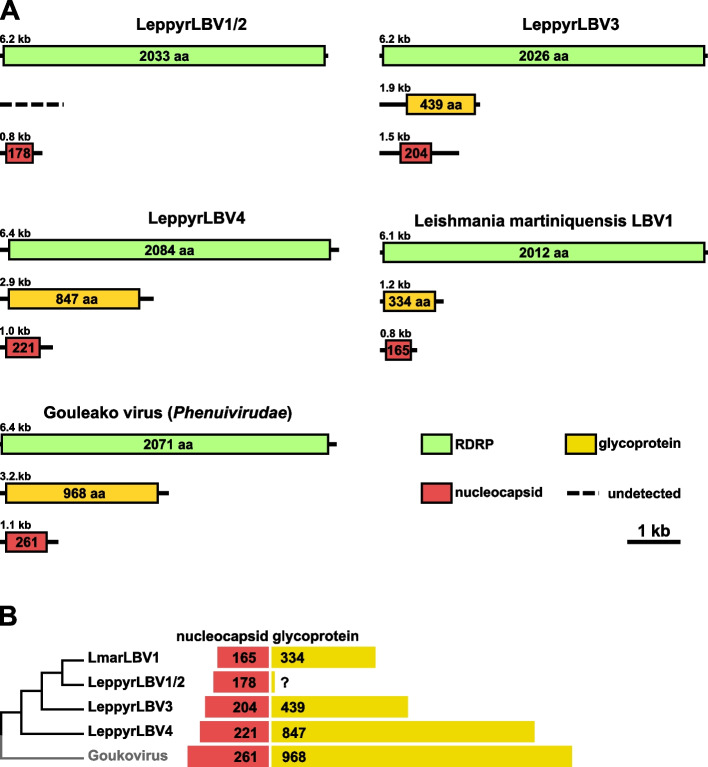
Genome variability of leishbuviruses from *Leptomonas pyrrhocoris*. **A** Schemes of genome organization of LeppyrLBVs and the reference species for the families Leishbuviridae and Phenuiviridae—Leishmania martiniquensis leishbuvirus 1 and Gouleako virus (*Goukovirus gouleakoense*), respectively. **B** Juxtaposition of schematic phylogeny and lengths of predicted nucleocapsid protein and glycoprotein demonstrating the gradual reduction of the latter in the evolution of Leishbuviridae

Similarly to other *Bunyavirales*, LBVs have three genomic segments: large (L), medium (M), and small (S) encoding the cap-snatching-endonuclease/RDRP complex, the glycoprotein, and the nucleoprotein, respectively [23]. We have previously demonstrated that the M and S segments of LBVs are much reduced in size as compared to those of the insect viruses from the sister family *Phenuiviridae* [14]. In LeppyrLBV1 and LeppyrLBV2, the S segment is 0.8 kb long, while the M segment could be identified neither on the gel nor in the NGS data (in the “crown” LBVs the respective sizes are 0.7–1.0 kb and 1.1–1.4 kb). Intriguingly, LeppyrLBV4, the most early-diverging representative of *L*. *pyrrhocoris* LBVs, contains the 2.9-kb-long M and 1.0-kb-long S segments (Fig. 5A, Additional file 1: Table S1), which are close in length to those found in Gouleako virus (*Goukovirus gouleakoense*) of the family *Phenuiviridae* (3.2 and 1.1 kb, respectively) [24]. In addition, clade 3 contains viruses with a 1.9-kb-long M segment (still longer than in LBVs described before) and a 1.5-kb-long S segment, which is even longer than that in all other related viruses considered here (Fig. 5A, Additional file 1: Table S1). The analysis of the proteins encoded in these segments (nucleocapsid and glycoprotein for M and S, respectively) revealed a clearer trend of gradual length reduction in the evolution of *Leishbuviridae* (Fig. 5B).

In one of the analyzed isolates, PL-Rz06, two distinct L segments of LeppyrLBV3 showing only 89.8% identity were assembled, while the minimal identity among all available sequences was 89.2%. A phylogenetic analysis of the nucleotide sequences of these segments demonstrated their association with two different clades within LeppyrLBV3 (Additional file 4: Fig. S1). This indicates the presence of two different strains of this virus in one isolate. Interestingly, this was not observable for the other two segments, apparently due to their lower variability (99.8% and 96.3% minimal identity for the M and S segments, respectively), which is counterintuitive considering that at the interspecific level, they are so divergent that it is sometimes difficult to reveal homology between them [14].

All bunyaviral segments have specific terminal complementary sequences (TCSs), which bring together both ends of genomic RNA forming a double-stranded “panhandle.” This facilitates RNA-RDRP interaction necessary for transcription, replication, and packaging of genomic segments into a virion. The TCS is a stretch of 20 to 30 complementary nucleotides interrupted by kinks or bulges [25]. In LeppyrLBVs, there are differences in sequence and shape of the TCSs not only between viruses, but also between the L, M, and S segments in different clades (Additional file 5: Fig. S2).

Showing the highest similarity of panhandles both in the primary and secondary structures, LBV4 represents an exception among these viruses. The most unusual is the L segment of LBV3, which contains a complex structure “disfiguring” the panhandle: a multi-branched loop, a big bulge, and a short hairpin (Additional file 5: Fig. S2). Such variability of TCSs revealed here is surprising. In *Bunyavirales*, the last eight nucleotides of TCS are conserved and family-specific [23]. For the L segment of the family *Phenuiviridae*, as well as LBV4 and all previously reported leishbuviruses, which form the crown group of LBVs in phylogenetic trees, this sequence is invariably ACACAAAG. The two sister species, LeppyrLBV1 and LeppyrLBV2, have a different sequence—AAG(A)AACA, while the related *Phytomonas* sp. TCC231 LBV1 has the canonical one [14]. In LBV3, that sequence is disrupted by the abovementioned complex structure (Additional file 5: Fig. S2).

Thus, the LBVs of *L. pyrrhocoris* represent both the missing link between the ancestral insect-infecting bunyaviruses similar to extant *Phenuiviridae*, as well as display significantly divergent TCSs. The basal phylogenetic position of *L*. *pyrrhocoris* LBVs can be explained by a restricted host range of *L*. *pyrrhocoris*, which infects predominantly firebugs [4, 26]. This resulted in the evolutionary preservation of the ancestral forms with the full-length M and S segments. The main driving force behind LBV diversification within *L*. *pyrrhocoris* appears to be a change in TCS, as clades 2, 3, and 4 deviate from the ancestral ACACAAAG form. The reasons for such changes, which independently occurred in three out of four *L. pyrrhocoris* LBVs, are obscure but may be related to the high prevalence and diversity of other viruses in this trypanosomatid species.

### Qin-like virus

**Fig. 6:**
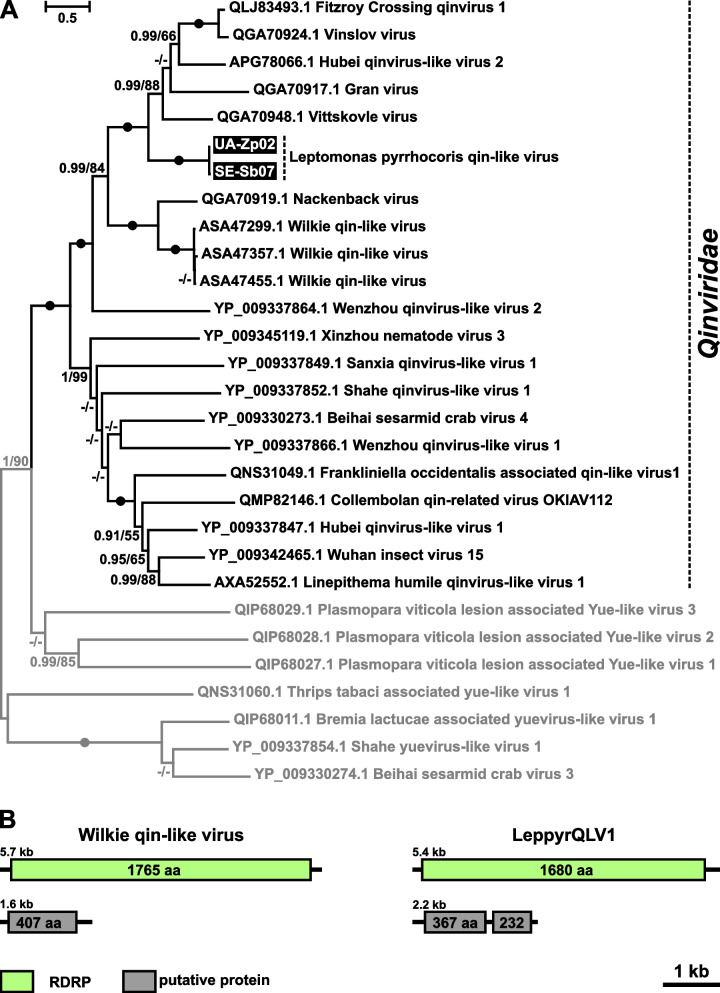
Qin-like virus of *Leptomonas pyrrhocoris*. **A** ML phylogenetic tree of Qinviridae based on RDRP amino acid sequences. Numbers at the branches indicate Bayesian posterior probability (PP) and ML bootstrap supports (BS). Only supports PP ≥ 0.8 and BS ≥ 50 are shown, lower values are replaced with dashes. Circles correspond to maximal statistical support by both methods. The scale bar indicates the number of substitutions per site. Outgroup is shown in gray. Inversed font and background colors indicate the strains studied in this work. **B** genomic organization of LeppyrQLV1 and its closest available reference—Wilkie qin-like virus

Two highly similar viruses detected in *L*. *pyrrhocoris* isolates from Ukraine and Serbia were identified as representatives of a single species of *Qinviridae*, a family of -ssRNA viruses recently discovered in metatranscriptomes of invertebrates [22]. It is considered that their genome consists of two segments, of which the first is 5–6.5 kb in size and encodes a large RDRP domain-containing protein, while the second one (in most members) is reported to be 1.6–1.9 kb long and code for a single hypothetical protein. Phylogenetically, Qinviridae along with a few other groups of bipartite viruses are close to non-segmented *Mononegavirales* and are united with them into the subphylum *Haploviricotina*, as opposed to multipartite *Polyploviricotina* [27]. The Leptomonas pyrrhocoris Qin-like virus (LeppyrQLV1) detected in this work falls into a clade together with viruses from metatranscriptomes of mosquitoes belonging to the genera *Aedes* and *Culex*, while other more distantly related representatives of the family were revealed in other arthropods and a nematode [28–30] (Fig. 6A). Notably, Qinviridae-specific reads in those metatranscriptomes had low abundance suggesting that these viruses infected rather microbiota than arthropods [28, 29].

Here, we documented the first representative of the family Qinviridae, for which the host specificity is unambiguously established. Similarly to other members of this group, its genome consists of two segments: 5.3 and 2.2 kb long (Fig. 6B). The large one encodes a 1679-aa-long protein with the RDRP domain. The small segment contains two ORFs in one frame coding for hypothetical proteins of 375 and 231 amino acids (aa) separated by a stop codon. This is distinct from the typical genome architecture for the family as exemplified by the closely related Wilkie qin-like virus, the small segment of which is shorter (1.6 kb) and comprises only a single ORF similar in size (407 aa) to the first one in LeppyrQLV1 (Fig. 6B). This longer ORF of LeppyrQLV1 yielded a few homologs from related meta-transcriptomic qinviruses, while nothing was retrieved for the shorter one from GenBank and UniClust20 databases. The larger hypothetical protein was predicted to be cytosolic and to contain two glycosylation sites, which is in line with an assumption that it is a nucleocapsid/matrix protein. In the shorter hypothetical protein, a single glycosylation site was identified and two transmembrane helices were predicted near the C-terminus, suggesting that it can be a surface glycoprotein. Interestingly, the distantly related Collembolan qin-related virus has been predicted to have these two proteins as well, but each of them resides in an individual genomic segment and their sequences are longer (623 and 430 aa for nucleocapsid and glycoprotein, respectively) [27].

### Analysis of virus distribution heterogeneity within isolates

The presence of more than one viral species in some isolates of *L. pyrrhocoris* raised a question of whether they represent mixtures of cells with different infection statuses. To address this question, we selected three cultures, in which two to four viruses had been detected: Lt-Vi09 (with LeppyrTLV1 and LeppyrLBV3), Pt-Li06 (with LeppyrTLV1 and LeppyrLBV3), and SE-Sb02 (with LeppyrTLV1, and LeppyrLBV2-LBV4). For each of these isolates, eight clones were analyzed by gel electrophoresis of dsRNAs (Additional file 6: Fig. S3).

In the Lt-Vi09 and PT-Li09 isolates, which bore mixtures of two viruses, individual clones displayed all possible variants of viral distribution. They could be virus-free, contain one of two viruses, or both of them at once. All analyzed clones of the SE-Sb02 isolate harbored LeppyrLBVs, of which we could reliably identify only LeppyrLBV2 and LeppyrLBV3 in some samples displaying the bands for the characteristic S segments (Additional file 6: Fig. S3). The absence of LeppyrTLV1 in the clones of SE-Sb02 is explained by the significantly lower proportion of this virus in the original uncloned isolate as compared to Lt-Vi09 and PT-Li09 (Additional file 6: Fig. S3).

The obtained results confirm our hypothesis on the mixed nature of *L. pyrrhocoris* isolates, being proxies of individual micropopulations of this species in the firebugs’ gut. It is very likely that with such a high parasite prevalence (up to 100% in some populations of *Pyrrhocoris apterus*) (Table 1), many individuals can be repeatedly infected by flagellates with different patterns of viral presence. Another important conclusion drawn from these findings is that two different viruses can coexist in a single cell. This resonates with our inferences suggesting coinfections of a single trypanosomatid cell by two strains of LeppyrTLV1 (see above).

Interestingly, some isolates can contain virus-free cells along with virus-bearing ones. Considering that the cultures underwent multiple passages, during which the cells had chances to become homogeneous in terms of viral infection status, two mutually non-exclusive explanations could be proposed: (1) some flagellates lose viruses due to inefficient segregation during cell division (vertical transmission) and/or (2) the horizontal transmission between cells is limited (e.g., due to insusceptibility of some cells or unsuitability of in vitro conditions for the viral exchange). It has been reported previously that viruses in trypanosomatid cultures can be stably preserved upon decades of continuous passaging or be depleted up to a complete loss [14]. The heterogeneity of cultures that we detected can explain the change in total viral load in the culture over time without the necessity to assume viral loss: the virus-free cells can overgrow the virus-bearing ones if they better fit to the in vitro conditions.

Little is known about the majority of the viral groups discussed here, but at least in LBVs, regular enveloped virions with glycoprotein spikes similar to those of Phenuiviridae have been detected by electron microscopy [14, 15]. Therefore, some viruses of *L. pyrrhocoris* can probably be shared through virion shedding. Another likely transmission route involves the exchange of extracellular vesicles, as demonstrated for LRV in *Leishmania* spp. [31, 32]. Finally, a direct cytoplasm contact occurring during cell mating can represent one more transmission route. Although the presence of genetic exchange requiring such a mechanism has not been so far demonstrated in *L. pyrrhocoris*, this is known to occur in its close relatives *Crithidia bombi* and *Leishmania* spp. [33–36].

## Conclusions

Our survey of RNA viruses in *L. pyrrhocoris* revealed seven viruses belonging to four groups. Only two out of these—LeppyrTLV1 (the most prevalent virus, present in 95.3% of all positive isolates or 57.5% of the tested ones) and LeppyrOV1 (12.5% and 7.5% prevalence, respectively)—have been previously detected in this flagellate. In addition, we discovered four new species of the family *Leishbuviridae* (20.3% and 12.3% prevalence, respectively), whose representatives have been previously documented in other trypanosomatids, and a new species of *Qinviridae* (3.1% and 1.9% prevalence, respectively), the family so far known only from the metatranscriptomes of invertebrates. Such a wide range of viruses, and the simultaneous presence of up to five different viruses in a single isolate of *L. pyrrhocoris*, are unprecedented among trypanosomatids. The uniqueness of this flagellate is determined by its peculiar host, the firebug *P. apterus.* This insect has a nearly cosmopolitan distribution and high abundance, ensuring the same for its parasites. Moreover, the gregarious lifestyle, coprophagy, and cannibalism of firebugs [37, 38] stipulate a very high (up to 100%) prevalence of *L. pyrrhocoris* [4]. This, in turn, creates conditions for repeated infections of the same host by parasites, which can represent different strains. Although most individuals of firebugs are short-winged and therefore flightless, there is also a long-winged (macropterous) morph, which can sometimes fly and is highly mobile in any case. This morph is considered to play the main role in the dispersal of this species [37, 39]. The active dispersal of firebugs creates conditions for mixing parasites from different host populations and, subsequently, mixing viruses, resulting in coinfection by different viral strains and species.

Importantly, *P. apterus* is polyphagous and, in addition to plant seeds, feeds on corpses of various invertebrates [37]. Thus, *L. pyrrhocoris* is exposed in the host intestine to a much wider spectrum of viruses than other trypanosomatids. After multiple attempts, some of these viruses can become adapted to this flagellate and eventually perform a host switch [40]. The representatives of *Leishbuviridae*, which are typical for trypanosomatids, could be adopted directly from other flagellates getting into the firebugs’ gut from the corpses of other insects. Conversely, *L. pyrrhocoris* probably cannot share its viruses with other trypanosomatids, since only this species and *Blastocrithidia papi* [41] are known to infect firebugs. In addition, the pungent defensive secretions of *P. apterus* repel the overwhelming majority of predators [37, 42], thereby restricting the chances of *L. pyrrhocoris* to “share” its viruses with trypanosomatids of other insects.

The multiplicity and high abundance of viruses in *L. pyrrhocoris* suggest that they may play a role in the relationship between this trypanosomatid and its firebug host. Although, to the best of our knowledge, this question has not been addressed so far for any trypanosomatid—insect pair, we can speculate based on the available information about the immune system in insects. In contrast to the majority of monoxenous trypanosomatids, *L. pyrrhocoris* was detected not only in the gut, but also in the hemolymph and salivary glands [43, 44]. The viruses released from infected flagellates via shedding, exocytosis, or following cell death can be perceived by the host immune system. The Toll pathway, known to play an important role in the biology of LRV-infected *Leishmania* spp., is also used by insects in antiviral and antimicrobial defense [45, 46]. Therefore, released viruses may trigger this pathway. The following activation of defense mechanisms such as recruitment of hemocytes to the infection sites (also including the gut) and melanization [47, 48] may lead to harsher conditions for the parasites, especially in the secondary (extraintestinal) infection sites. While frequent gut infections do not seem to be affected by viruses (Table 1), this may not be the case in rather rare infections of hemolymph and salivary glands [49].

The effects of viruses described here can be very diverse. Indeed, even closely related viral species can have different levels of integration into the cellular processes of their trypanosomatid hosts. In a recent experimental study, the removal of LRV1 from *Leishmania guyanensis* had no significant effect on the growth and transcription profile of the latter, whereas ablation of LRV2 from *L. major* resulted in a decreased proliferation rate and conspicuous stress effect as judged by changes in the gene expression [50].

In sum, the peculiar biology of the insect host makes *L. pyrrhocoris* a unique “hoarder” of viruses collected from various sources. We propose that this flagellate is a good model to study the adoption of new viruses and their relationships with a protist host. Since our study concerned only European isolates, the global diversity of viruses in this trypanosomatid is likely to be significantly higher.

## Methods

### Collection, cultivation, and identification of isolates

The screening for the presence of dsRNA included 106 trypanosomatid isolates established from field samples of 508 firebugs (*Pyrrhocoris apterus*) from 13 European countries (Table 1). Insects were dissected and analyzed for the presence of trypanosomatids as described previously [51]. Cultured parasites were maintained in the Brain Heart Infusion medium (Sigma-Aldrich/Merck, St. Louis, USA) supplemented with 10 µg/ml of hemin (Jena Bioscience, Jena, Germany), 10% fetal bovine serum (FBS), 500 units/ml of penicillin, and 0.5 mg/ml of streptomycin (all from Thermo Fisher Scientific, Waltham, USA). DNA for the species validation was isolated from 5 × 10^7^ cells using the Qiagen DNeasy Blood & Tissue kit (Qiagen, Hilden, Germany). All isolates were confirmed to be axenic *L. pyrrhocoris* by 18S rRNA and glycosomal glyceraldehyde phosphate dehydrogenase gene sequence analyses as described previously [52].

### RNA isolation, dsRNA purification, and next-generation sequencing

Total RNA was isolated from 5 × 10^8^ to 1 × 10^9^ cells using the TRI Reagent (Molecular Research Center, Cincinnati, USA) as described elsewhere [53]. For the initial screening, 50 µg of total RNA was treated with DNase I/S1 nuclease enzyme mix [54, 55]. The resulting dsRNA was resolved on 0.8% agarose gel and post-stained with Midori green dye (Nippon Genetic Europe, Düren, Germany). For next-generation sequencing (NGS), 400 µg of total RNA was digested with DNase I/S1 nuclease enzyme mix and purified using the Zymoclean Gel RNA recovery kit (Zymo Research, Irvine, USA). The RiboMinus libraries were sequenced using Illumina HiSeq 2500 (Illumina, San Diego, CA, USA) at Macrogen Inc. (Amsterdam, The Netherlands) or the Institute of Applied Biotechnologies (Olomouc, Czechia).

### Virus genome assembly and search

Reads were trimmed using Trimmomatic v. 0.40 [56] (ILLUMINACLIP:TruSeq3-PE-2.fa:2:20:10 LEADING:3 TRAILING:3 SLIDINGWINDOW:4:15 MINLEN:50) and assembled de novo with Trinity v. 2.13.2 [57]. The mapping and sorting of reads were performed using Bowtie 2 v. 2.4.4 [58] and SAMtools v.1.13 [59], respectively, with default settings. The read per kilobase per million (RPKM) values for each sample were calculated using a custom awk script from the “per base” coverage file generated by BEDTools v. 2.30.0 [60]. Contigs containing viral genes were identified by BLASTN (BLAST + v. 2.12 [61]) and BLASTX (DIAMOND v. 2.0.2 [62]) searches against UniClust50 database. Nucleocapsid proteins of divergent leishbuviruses were found by HHblits search [63] against a custom-built sequence profile of nucleocapsids from Leishbuviridae and Phenuiviridae. Additional viral ORFs were identified using the following criteria: (i) contig length corresponding to that of the dsRNA band on the gel, (ii) contig coverage correlating with the relative brightness of the dsRNA band as compared to an identified contig/band pair, and (iii) the presence of specific viral terminal sequences. The analysis of putative Qin-like virus glycoprotein was performed with web-based tools: CCTOP [64] for transmembrane helices, NetNGlyc v. 1.0 [65] for N-linked glycosylation sites, and SignalP v. 6.0 [66] for signal peptides. Assembled viral fragments were submitted to GenBank under the accession numbers OP722764 – OP722922.

### Phylogenetic inferences

For LBVs, the dataset of RNA-dependent RNA polymerase (RDRP) proteins was taken from [15]. Homologs of divergent LBVs were added by running BLASTP [61] with the respective sequences against the non-redundant (nr) database of GenBank. The sequences were aligned in MAFFT v. 7.490 [67] using the G-INS-i algorithm with a maximum of 1000 iterations and trimmed in TrimAl v. 1.4 [68] with “automated1” algorithm resulting in 1139 amino acid positions. The maximum likelihood (ML) phylogenetic tree was inferred in IQ-Tree v. 2.2.0 under the automatically selected LG + I + G4 + F substitution model and branch supports estimated using 1000 thorough bootstrap replicates [69]. The tree was rooted according to the topologies obtained in the previous studies using wider taxonomic datasets [14, 16]. Bayesian inference of phylogeny was performed in MrBayes v. 3.2.7 run for 1 million generations under the same substitution model as above, sampling every 250th generation and all other parameters set by default [70]. Posterior probability values from Bayesian analysis were overlaid onto the ML tree topology with bootstrap supports.

A similar approach was applied to novel Qin-like viruses. The sequences of RDRP homologs were retrieved from the nr database using PSI-BLAST (4 iterations), and only Qin-like viruses and their closest relatives—Yue-like viruses (serving as outgroup)—were taken for further analysis. Sequences were aligned in MAFFT using the E-INS-i algorithm with default settings and trimmed as above. The resulting alignment of 1047 amino acid positions was analyzed as above with the only difference being that the Bayesian analysis was run for 1.5 million generations.

For the inference of the phylogenetic position of LeppyrTLV1, the amino acid RDRP sequences of 32 tombus-like viruses and 32 outgroups, belonging to chronic bee paralysis virus (CBPV)-like viruses, Alpha-like viruses, and Noda-like viruses were retrieved from the GenBank, and processed as for Qin-like viruses, resulting in a 255-amino acid (aa)-long alignment. The ML and Bayesian inferences were performed as above except for the latter analysis which was run for 4 million generations.

Phylogenetic relationships between the strains of LeppyrTLV1 were inferred using sequences obtained in this work and those for the isolates H10 and F165 published previously [14]. Analyses were performed separately for each of the three ORFs using automatically selected nucleotide substitution models with partitioning by codon position in IQ-Tree and MrBayes with rate multiplier unlinked across partitions. The significance of the discordance between individual ORF topologies was assessed using the likelihood ratio test and Bayes factors [71]. For that, phylogenetic inferences with linked and unlinked topologies for the three ORFs were performed using nucleotide and amino acid substitution models in both IQ-Tree and MrBayes. The marginal likelihoods for the Bayes factors were estimated using the stepping stone method [70]. The *p*-values for the *χ*^2^ statistic obtained in LRT analysis were calculated using an online tool at https://goodcalculators.com/chi-square-calculator/.

For the reconstruction of phylogenetic relationships between ostraviruses, the sequences of all seven ORFs were concatenated after processing as for the qinviruses using a custom bash script. The ML and Bayesian inferences were performed without partitioning under the GTR + F + I model and other details as for LBVs.

### Analysis of virus distribution heterogeneity within isolates

Considering that the isolates of *Leptomonas pyrrhocoris* obtained in this work could represent mixtures of cells differing in viral infection status, we performed an additional screening of viruses on the clonal level. For that, we selected three isolates that showed the presence of more than one virus on the gel: Lt-Vi09, PT-Li06, and Se-Sb02. They were cloned by serial dilution in microtiter plates (0.2 cells per well) and eight clones in each case were screened by gel electrophoresis of dsRNA preparations as described above.

### Supplementary Information

**Additional file 1: Table S1.** Summary of the NGS data obtained for the viral RNAs of the studied trypanosomatid isolates.

**Additional file 2: Table S2. **Sequence variation in LeppyrTLV1 and LeppyrOV1.

**Additional file 3: Table S3. **Analysis of the incompatibility of tree topologies for different ORFs in LeppyrTLV1.

**Additional file 4: Fig. S1.** Phylogenetic inference of relationships between the RDRP nucleotide sequences of LeppyrLBV3. Numbers at the branches indicate Bayesian posterior probability (PP) and ML bootstrap supports (BS), respectively. Only bootstrap supports BS ≥ 50 are shown, lower values replaced with dashes (-). Circles correspond to maximal statistical support by both methods. The scale bar indicates the number of substitutions per site.

**Additional file 5: Fig. S2. **Terminal complementary sequences in LBVs of *Leptomonas pyrrhocoris*. Primary structures and/or alignments are shown on the left, secondary structures are on the right. The panhandle-distorting complex structure containing a multi-branched loop, a big bulge, and a short hairpin is outlined.

**Additional file 6: Fig. S3. **Analysis of virus distribution heterogeneity within isolates. Note that not all fragments for a virus (see graphic legend) can be always detected on the gel.

## Data Availability

The sequences obtained in this study are available from the GenBank under the accession numbers OP722764 – OP722922.

## Abbreviations

BS: Bootstrap support
CBPV: Chronic bee paralysis virus
CQRV: Collembolan qin-related virus
dsRNA: Double-stranded RNA
L (segment): Large
LBV(s): Leishbuvirus(es), Leishbuviridae
LeppyrLBV: Leptomonas pyrrhocoris Leishbuvirus
LeppyrOV1: Leptomonas pyrrhocoris Ostravirus 1
LeppyrTLV1: Leptomonas pyrrhocoris tombus-like virus 1
LRV: Leishmania RNA virus
M (segment): Medium
ML: Maximum likelihood
NGS: Next-generation sequencing
ORF: Open reading frames
PP: Posterior probability
RDRP: RNA-dependent RNA polymerase
RPKM: Read per kilobase per million
S (segment): Small
-ssRNA: Negative-sense single-stranded RNA
TCSs: Terminal complementary sequences
TLV: Tombus-like virus(es)
