# Supplementary figures and images for: Diversity of RNA viruses in the cosmopolitan monoxenous trypanosomatid Leptomonas pyrrhocoris
Source: BMC Biol. 2023 Sep 12;21:191. doi: 10.1186/s12915-023-01687-y (PMC10496375; doi:10.1186/s12915-023-01687-y)

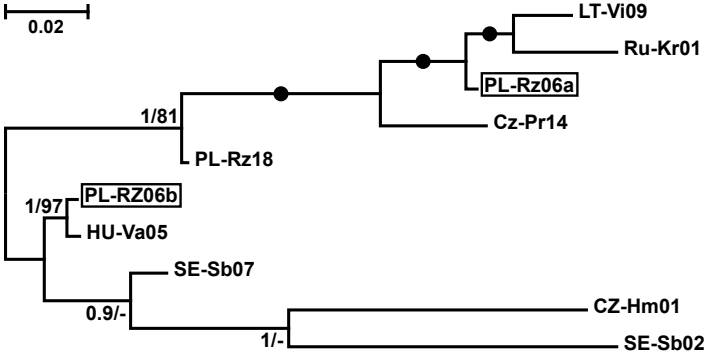

Supplement: Supplementary file 4 — Additional file 4: Fig. S1. Phylogenetic inference of relationships between the RDRP nucleotide sequences of LeppyrLBV3. Numbers at the branches indicate Bayesian posterior probability (PP) and ML bootstrap supports (BS), respectively. Only bootstrap supports BS ≥ 50 are shown, lower values replaced with dashes (-). Circles correspond to maximal statistical support by both methods. The scale bar indicates the number of substitutions per site. [file 12915_2023_1687_MOESM4_ESM.pdf]

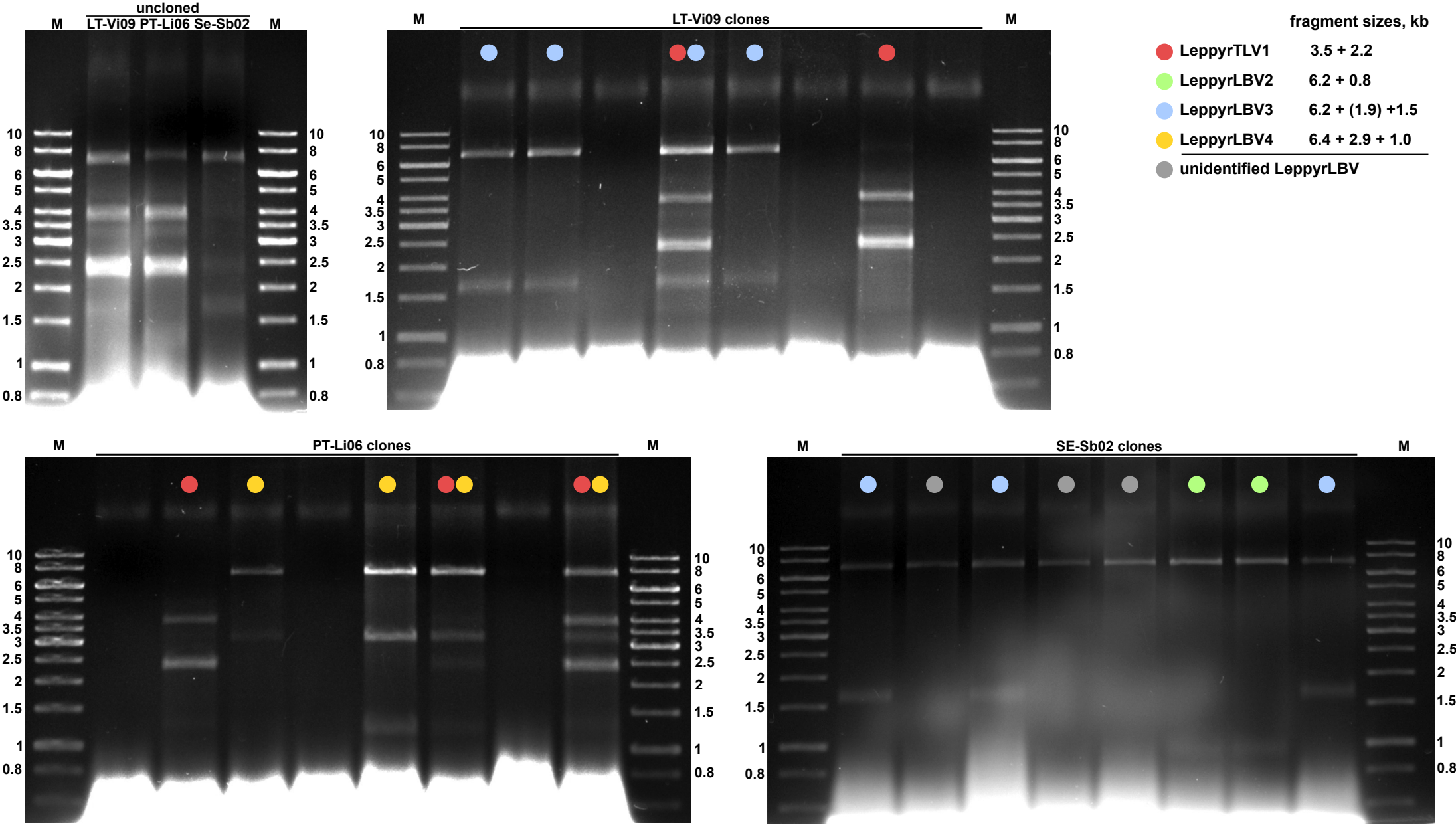

Supplement: Supplementary file 6 — Additional file 6: Fig. S3. Analysis of virus distribution heterogeneity within isolates. Note that not all fragments for a virus (see graphic legend) can be always detected on the gel. [file 12915_2023_1687_MOESM6_ESM.pdf]
