# Supplementary material for: Diversity of RNA viruses in the cosmopolitan monoxenous trypanosomatid Leptomonas pyrrhocoris
Source: BMC Biol. 2023 Sep 12;21:191. doi: 10.1186/s12915-023-01687-y (PMC10496375; doi:10.1186/s12915-023-01687-y)
Supplement: Supplementary file 5 — Additional file 5: Fig. S2. Terminal complementary sequences in LBVs of Leptomonas pyrrhocoris. Primary structures and/or alignments are shown on the left, secondary structures are on the right. The panhandle-distorting complex structure containing a multi-branched loop, a big bulge, and a short hairpin is outlined. [file 12915_2023_1687_MOESM5_ESM.pdf]

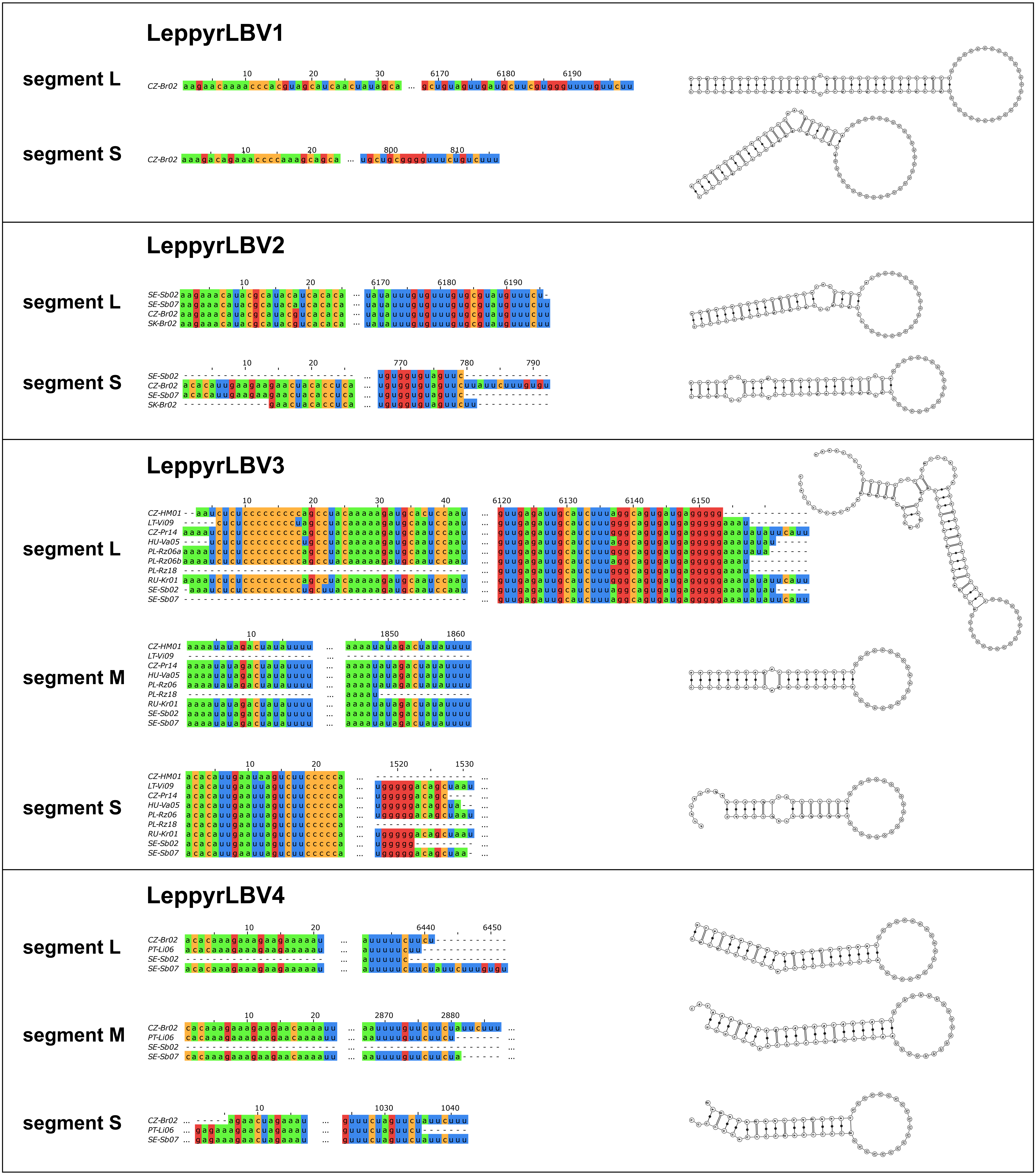

LeppyrLBV1
segment L
CZ-Br02
segment S
CZ-Br02
LeppyrLBV2
segment L
SE-Sb02
SE-Sb07
CZ-Br02
SK-Br02
segment S
SE-Sb02
CZ-Br02
SE-Sb07
SK-Br02
LeppyrLBV3
segment L
CZ-HM01
LT-Vi09
CZ-Pr14
HU-Va05
PL-Rz06a
PL-Rz06b
PL-Rz18
RU-Kr01
SE-Sb02
SE-Sb07
segment M
CZ-HM01
LT-Vi09
CZ-Pr14
HU-Va05
PL-Rz06
PL-Rz18
RU-Kr01
SE-Sb02
SE-Sb07
segment S
CZ-HM01
LT-Vi09
CZ-Pr14
HU-Va05
PL-Rz06
PL-Rz18
RU-Kr01
SE-Sb02
SE-Sb07
LeppyrLBV4
segment L
CZ-Br02
PT-Li06
SE-Sb02
SE-Sb07
segment M
CZ-Br02
PT-Li06
SE-Sb02
SE-Sb07
segment S
CZ-Br02
PT-Li06
SE-Sb07
